# Effect and safety of 4% albumin in the treatment of cardiac surgery patients: study protocol for the randomized, double-blind, clinical ALBICS (ALBumin In Cardiac Surgery) trial

**DOI:** 10.1186/s13063-020-4160-3

**Published:** 2020-02-28

**Authors:** Hanna Vlasov, Tatu Juvonen, Seppo Hiippala, Raili Suojaranta, Markku Peltonen, Alexey Schramko, Kaapo Arvonen, Ulla-Stina Salminen, Ilona Kleine Budde, Tiina Eränen, Maxim Mazanikov, Mihkel Meinberg, Tommi Vähäsilta, Erika Wilkman, Ville Pettilä, Eero Pesonen

**Affiliations:** 1 Department of Anesthesiology and Intensive Care and Pain Medicine, University of Helsinki and Helsinki University Hospital, Helsinki, Finland; 2 Department of Cardiac Surgery, Heart and Lung Center, University of Helsinki and Helsinki University Hospital, Helsinki, Finland; 3 National Institute for Health and Welfare, Helsinki, Finland; 4 Department of Clinical Operations, Sanquin Plasma Products B.V., Amsterdam, The Netherlands; 5 HUS Pharmacy, University of Helsinki and Helsinki University Hospital, Helsinki, Finland

**Keywords:** Albumin, Ringer’s acetate, Cardiac surgery, Cardiopulmonary bypass, Fluid management

## Abstract

**Background:**

In cardiac surgery with cardiopulmonary bypass (CPB), large amounts of fluids are administered. CPB priming with crystalloid solution causes marked hemodilution and fluid extravasation. Colloid solutions may reduce fluid overload because they have a better volume expansion effect than crystalloids. The European Medicines Agency does not recommend the use of hydroxyethyl starch solutions (HES) due to harmful renal effects. Albumin solution does not impair blood coagulation but the findings on kidney function are conflicting. On the other hand, albumin may reduce endothelial glycocalyx destruction and decrease platelet count during CPB. No large randomized, double-blind, clinical trials have compared albumin solution to crystalloid solution in cardiac surgery.

**Methods/design:**

In this single-center, double-blind, randomized controlled trial comprising 1386 adult cardiac surgery patients, 4% albumin solution will be compared to Ringer’s acetate solution in CPB priming and volume replacement up to 3200 mL during surgery and the first 24 h of intensive care unit stay. The primary efficacy outcome is the number of patients with at least one major adverse event (MAE) during 90 postoperative days (all-cause death, acute myocardial injury, acute heart failure or low output syndrome, resternotomy, stroke, major arrhythmia, major bleeding, infection compromising post-procedural rehabilitation, acute kidney injury). Secondary outcomes are total number of MAEs, incidence of major adverse cardiac events (MACE; cardiac death, acute myocardial injury, acute heart failure, arrhythmia), amount of each type of blood product transfused (red blood cells, fresh frozen plasma, platelets), total fluid balance at the end of the intervention period, total measured blood loss, development of acute kidney injury, days alive without mechanical ventilation in 90 days, days alive outside intensive care unit at 90 days, days alive at home at 90 days, and 90-day mortality.

**Discussion:**

The findings of this study will provide new evidence regarding efficacy and safety of albumin solution in adult patients undergoing cardiac surgery with CPB.

**Trial registration:**

EudraCT (clinicaltrialsregister.eu) 2015–002556-27 Registered 11 Nov 2016 and ClinicalTrials.gov NCT02560519. Registered 25 Sept 2015.

## Background

In cardiac surgery, intravenous fluid resuscitation is crucial to ensure adequate intravascular volume, stroke volume, and tissue oxygen delivery. At the same time, prevention of fluid overload is essential. Acute hypervolemia causes endothelial glycocalyx destruction and fluid shift to the interstitial space, leading to postoperative complications and increased mortality [1–3]. Hemodilution increases the use of packed red blood cell (PRBC) transfusion, which increases morbidity and mortality after cardiac surgery [4]. In the literature, controversy exists about the optimal perioperative fluid management in patients undergoing cardiac surgery.

Fluid management in cardiac surgery differs from fluid management in other types of major surgery. As a central pathophysiological phenomenon of cardiopulmonary bypass (CPB), the systemic inflammatory response leads to damage of the endothelial glycocalyx, capillary hyperpermeability, microcirculatory dysfunction, and reduced tissue oxygenation [5, 6]. CPB priming with crystalloid solutions causes marked hemodilution, decreased colloid osmotic pressure, and increased total body water content. As a combined consequence of hyperpermeability and hemodilution, most patients present with fluid overload after CPB. In addition, cardioplegia, deliberate hypothermia, ischemia-reperfusion injury, systemic heparinization, other coagulation disturbances, microemboli formation, and high doses of vasoactive and inotropic drugs affect fluid status [7, 8]. The complexity of hemodynamic impairment occurring during and after cardiac surgery leads to difficulties in assessing the actual volume status.

No consensus on the optimal fluid therapy in cardiac surgery exists. Of note, endothelial glycocalyx destruction may change the dynamics of colloids and crystalloids. Crystalloid solutions affect neither renal function nor blood coagulation more than hemodilution could explain. Colloid solutions, due to their volume expansion capacity, maintain intravascular volume better than crystalloids. After major surgery, maintenance of adequate cardiac preload requires almost twice as much volume of crystalloids as that of colloids [9]. Thus, colloids may be more suitable for restrictive fluid therapy in cardiac surgery. Hydroxyethyl starch (HES) solutions have previously been used for volume expansion in cardiac surgery. However, two large randomized clinical trials (RCT) showed harmful effects of rapidly degradable HES solutions on renal function in critically ill patients [10, 11].

In cardiac surgery, albumin solution has been demonstrated to maintain hemodynamics as well as HES solutions [12, 13] and better than crystalloids [14]. Albumin solutions do not impair coagulation [15]. Although albumin solution is considered safe with regard to kidney function, the existing data are conflicting [16]. In a retrospective cohort study using a propensity score methodology, albumin administration was associated with a dose-dependent risk of acute kidney injury (AKI) [17]. In another retrospective cohort study on on-pump cardiac surgery, the use of 5% albumin solution was associated with significantly decreased odds of in-hospital mortality and all-cause 30-day readmission rate compared with administration of crystalloids alone [18]. In a small randomized trial on on-pump cardiac surgery (76–81 patients/group), fluid management with albumin resulted in less positive fluid balance compared to HES and Ringer’s lactate. However, creatinine levels were significantly higher in the early postoperative phase in the albumin group, compared to Ringer’s lactate and HES groups [19]. On the other hand, in a randomized controlled trial on off-pump coronary artery bypass surgery (110 patients/group), preoperative correction of hypoalbuminemia with exogenous albumin protected from acute kidney injury [20].

Glycocalyx regulates vascular permeability and inflammation and coagulation on the endothelial surface. Sphingosine-1-phosphate (S1P) in plasma regulates the synthesis and degradation of glycocalyx [21]. Furthermore, S1P modulates continuity of tight junctions, another factor related to vascular permeability. Serum albumin is essential for the bioavailability of S1P [22]. Albumin not only triggers the release of S1P from red blood cells, the main reservoir of S1P in blood, but also carries it to S1P-receptors on endothelial cells. Activation of S1P-receptor 1 on endothelial cells inhibits matrix metalloproteinase 9 and matrix metalloproteinase 13 dependent shedding of syndecan-1 ectodomain, an important component of glycocalyx [23]. Removal of plasma proteins from the culture media results in destruction of glycocalyx [23]. Conversely, in experimental heart transplantation, addition of albumin into the storage solution preserves glycocalyx of the coronary circulation [24]. If the CPB circuit is primed only with crystalloid solution, initiation of CPB will result in prompt and profound hypoalbuminemia.

No large RCTs comparing albumin solution to crystalloid solution in cardiac surgical patients have been conducted. Herein, we describe the final protocol (version 5, 3 September, 2018) for a randomized, parallel-group, double-blind study comparing 4% albumin solution to Ringer’s acetate solution in cardiac surgery with CPB. We hypothesize that CPB priming and intravascular volume replacement therapy using 4% albumin causes less major adverse events (MAE) than Ringer’s acetate solution.

The article is written in accordance with the Standard Protocol Items: Recommendations for Interventional Trials (SPIRIT) guidelines. The SPIRIT checklist is provided as Additional file 1.

## Methods/design

### Patients

This study will be a double-blind, randomized, controlled trial in 1386 cardiac surgery patients scheduled for elective surgery or operated on during the index admission in Helsinki University Hospital, Helsinki, Finland. The patients will be allocated 1:1 into the two study groups.

#### Inclusion criteria

- Aged 18–90 years
- Primary or repeat open heart surgery procedures, either independently or in combinations
  - ▪ Coronary artery bypass grafting
  - ▪ Aortic valve replacement/repair
  - ▪ Mitral valve replacement/repair
  - ▪ Tricuspid valve replacement/repair
  - ▪ The maze procedure or its modifications
  - ▪ Aortic root or ascending aorta surgery when no hypothermic circulatory arrest is required
- Scheduled for elective surgery or operated on during the index admission

#### Exclusion criteria

- Immediate emergency surgery (i.e., no time for recruitment)
- Correction of a congenital cardiac defect
- Preoperative infection compromising post-procedural rehabilitation
- Preoperative heart failure/low output sdr, defined as preoperative inotropic support, mechanical assistance of breathing, preoperative extracorporeal membrane oxygenation (ECMO) support, preoperative intra-aortic balloon pump, preoperative mechanical assistance of left ventricle, preoperative ejection fraction (EF) < 20% (intraoperative transesophageal echocardiography not included), some other comparable preoperative conditions; the condition has to be ongoing
- End-stage kidney disease (estimated glomerular filtration rate (GFR) < 20 mL/min, based on serum/plasma creatinine)
- Hemophilia A, hemophilia B
- Patient refusal of the use of blood products and derivatives of blood products
- Ticagrelol, prasugrel, or clopidogrel treatment within 2 days
- Apixaban or rivaroxaban treatment within 2 days or dabigatran treatment within 3 days

### Intervention

The trial will be an intervention in patients undergoing cardiac surgery with CPB between two study solutions: albumin solution at a final concentration of 4% and Ringer’s acetate solution. The trial consists of two phases. In both phases the same study solution (albumin or Ringer’s acetate) will be used in a double-blind fashion (Table 1). In the first phase of the trial, the study solution will be used for priming of the CPB circuit. In the second phase of the trial, during surgery and during the first 24 h in the intensive care unit (ICU), study solution will be used as volume replacement therapy up to 3200 mL. If more than 3200 mL of volume replacement is needed during the second study phase, Ringer’s acetate solution will be used thereafter. The volume replacement therapy will not be set by protocol and will be based on the clinical decision.

**Table 1:** Trial design for ALBumin In Cardiac Surgery (ALBICS) study

Study intervention	Albumin group: study solution	Ringer group: study solution
Unblinded CPB priming Blinded CPB priming	1200 mL of Ringer’s acetate 300 mL of 20% albumin (final albumin concentration of 4%)	1200 mL of Ringer’s acetate 300 mL of Ringer’s acetate (pure Ringer’s acetate)
Blinded volume replacement up to 3200 ml^a^	Albumin 4%	Ringer’s acetate
Unblinded volume replacement over 3200 ml^a^	Ringer’s acetate	Ringer’s acetate
Other fluid management		
Background infusion^a^	Ringer’s acetate 0.5 mL/kg/h	Ringer’s acetate 0.5 mL/kg/h
Blood transfusions^a^	Based on clinical decision, excluding albumin	Based on clinical decision, excluding albumin

^a^During surgery and the first 24 h in ICU

#### Albumin group

- The first phase: The CPB circuit will be initially primed with 1200 mL of Ringer’s acetate solution in an unblinded manner. Thereafter, in a blinded manner, 300 mL of 20% (200 g/L) albumin solution (Albuman® 200 g/L, Sanquin Plasma Products BV, the Netherlands) will be added, resulting in a final albumin concentration of 4% in the priming fluid.
- The second phase: 4% (40 g/L) albumin solution (Albuman®, Sanquin Plasma Products BV, the Netherlands) in a blinded manner.

#### Ringer group

- The first phase: The CPB circuit will be initially primed with 1200 mL of Ringer’s acetate solution in an unblinded manner. Thereafter, in a blinded manner, 300 mL of Ringer’s acetate solution (RingerAcetat Baxter Viaflo®, Baxter, Finland) will be added, resulting in pure Ringer’s acetate solution as the priming fluid.
- The second phase: Ringer’s acetate solution (RingerAcetat Baxter Viaflo®, Baxter, Finland) in a blinded manner.

**Fig. 1:**
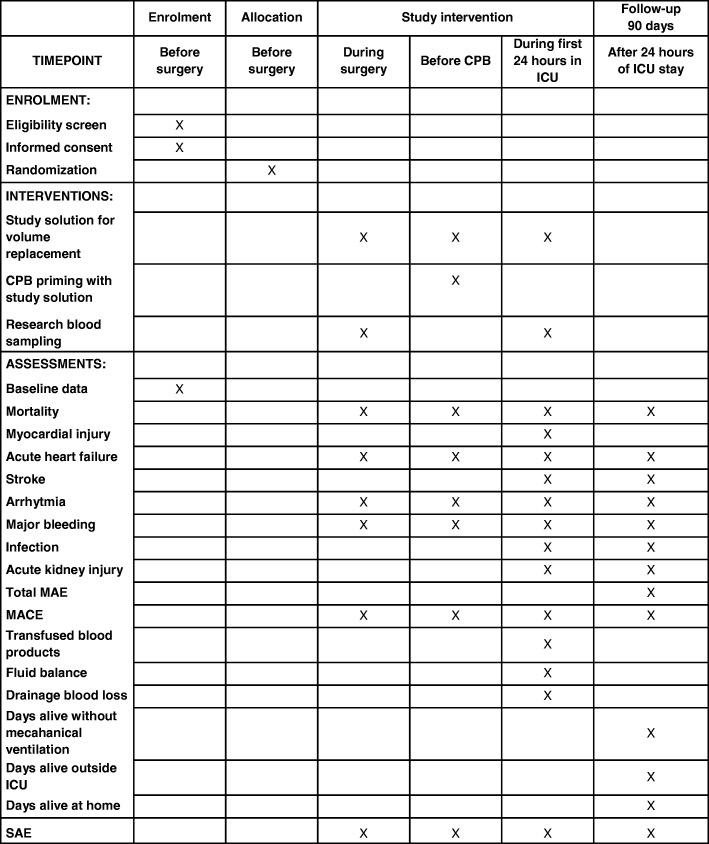
Schedule of procedures. ^a^Blood samples will be collected at the following time points: after placement of the arterial cannula before induction of anesthesia, 30 min after protamine administration, 6 h after aortic declamping, and 20 ± 1 h after aortic declamping

The intervention period will end after the first 24 h of ICU stay or when the patient leaves the ICU if the latter occurs within the first postoperative 24 h (Fig. 1.) After the intervention period, fluids will be administered according to local clinical practice.

For physiological basic need of fluid, Ringer’s acetate solution will be given throughout the intervention period as a background infusion of 0.5 mL/kg/h, rounded to the nearest multiple of 10 mL.

### Concomitant treatment

Apart from the study intervention, patients will be treated according to the clinical practice of the hospital. This includes transfusion of blood products, except albumin. CPB will be performed using a non-pulsatile pump and a membrane oxygenator in a standard manner. After protamine administration shed mediastinal blood will be discarded. According to clinical judgment, the whole CPB residual volume or part of it may be re-transfused after weaning from CPB. All drugs (antibiotics, vasoactive agents, electrolyte concentration, etc.) will be dissolved in either 0.9% NaCl or 5% glucose solution according to the prevailing clinical practice. All drug solutions will be counted in the fluid balance.

### Randomization

Randomization will be done every other week at HUS Pharmacy (the hospital pharmacy of Helsinki University Hospital) using online software (www.randomization.com) in conjuction with the production of the blinded study solution bags. Only the personnel of HUS Pharmacy, who will not take part in patient care, will be aware of the randomization. Consequently, the entire study group, including the study nurse, and the personnel taking care of the patients will be kept blinded to randomization. HUS Pharmacy will deliver a set of nine study solution bags for each patient (see below). For each set, HUS Pharmacy will give a unique consecutive allocation number. Randomization of consecutive allocation numbers into the two study arms will be performed in blocks before preparation of each lot of study solution bag sets. One randomized block is equivalent to one produced lot of study solution bag sets and their respective allocation numbers. The actual patient randomization will occur preoperatively when the next available study solution bag set for the subsequent study patient will be taken into use (see below). The allocation number of the study solution bag set will be the patient’s study number. Varying block sizes will be used taking into account reductions during public holidays. The block sizes will be chosen to match the estimated patient recruitment rate within the shelf life of the study solution bags (see below). Preferentially, the block sizes will be 24 patients and 30 patients, alternating every other preparation lot. Other block sizes may be used if needed. In the blocks, patients will be randomized to the two treatment groups in a 1:1 ratio, except for the last few blocks of the study. Patient withdrawal and expiration of study solution bag sets may imbalance the patient allocation. In order to compensate for potential imbalance, patients may be randomized unequally in the last few blocks to retain the intended ratio of 1:1 between the study groups in all study patients.

### Study solutions and blinding

#### Preparation and disposal of blinded study solution bags

Preparation, packing, and labeling of the blinded study solution bags will be done in HUS Pharmacy according to the requirements of Current Good Manufacturing Practises (cGMP) and the International Council for Harmonisation Good Clinical Practise (ICH-GCP) guidelines. Commercial products Albuman® 200 g/L and Albuman® 40 g/L are packed in glass bottles and Ringer-Acetat Baxter Viaflo® in plastic bags. Albumin solution is slightly viscous and has an easily visible yellow color while Ringer’s acetate is non-viscous and colorless. To ensure adequate blinding, both study solutions will be packed in identical ethyl vinyl acetate (EVA) 500 mL bags (EVA Parenteral Nutrition Container, Baxter), which have been tested to be biocompatible for study solutions for at least 28 days and sterile. EVA bags will be placed in non-transparent cover bags (Opaque Protection Bag for Light Sensitive Drugs, Maco Pharma, Germany) labeled with blinded patient-specific labels. For each patient, HUS Pharmacy will deliver a set of nine study solution bags: one bag (300 mL) for the first phase of the trial and eight bags (400 mL/bag) for the second phase. The shelf life of the study solution bags will be 28 days, which will leave a 21-day usage time in addition to a 7-day period reserved for their preparation. After the intervention period, the personnel of the Reprocessing Unit for Medical Devices, which does not take part in patient care, will dispose of both the used and unused study solution bags.

#### Blinding during delivery of study solutions

Preoperatively, consecutive study patients will be given the first available set of blinded study solution bags. Each set will be labeled with a unique allocation number, which will also be the patient’s study number. The set contains nine blinded study solution bags: one bag (300 mL) for the first phase of the trial and eight bags (400 mL/bag) for the second phase (see above). In the first phase of the trial, the CPB circuit will initially be primed with 1200 mL of Ringer’s acetate solution. To ensure adequate blinding, after systemic heparinization, a small amount of blood will be drawn from the operation field to the CPB reservoir to make the initial priming fluid of Ringer’s acetate blood-colored. For the blinding, the tube used for filling the CPB reservoir will be covered with non-transparent adhesive tape to mask the viscosity and color of the albumin solution. Thereafter, the study solution bag of 300 mL will be added to the initial priming fluid. This will result in a total priming volume of 1500 mL containing either albumin solution at a final concentration of 4% (Albumin group) or pure Ringer’s acetate solution (Ringer group). In the second phase of the trial, the study solution will be administered using colored but transparent infusion tubes (B Braun, Infusomat). Because albumin solution is slightly viscous, which may be noticed on visual inspection, the drop chamber will be covered with non-transparent adhesive tape and tubes will be prefilled with saline. Study solutions will be administered with infusion pumps.

### Outcomes

The follow-up period for all primary and secondary outcome measures will be 90 postoperative days.

#### Primary outcome measure

The primary efficacy endpoint will be the proportion of patients with at least one MAE during the study period of 90 days (Fig. 1). The composite end-point of MAE was adopted with small modifications from a previous publication [25].

MAE composition

- All-cause death
- Acute myocardial injury (defined as an increase in creatinine kinase muscle/brain isoenzymes (CK-MB) at least ten times as high as the upper normal limit of the local laboratory, i.e., 70 μg/l)
- New onset of acute heart failure (e.g., pulmonary edema, cardiogenic shock) or low output syndrome requiring intravenous inotropic agents and/or intra-aortic balloon pump support, ECMO support, or other comparable condition
- Resternotomy, subxiphoidal pericardial drainage, or other comparable postoperative procedure
- Stroke
- Major arrhythmia (ventricular fibrillation off-CPB, ventricular tachycardia off-CPB, new onset atrial fibrillation of permanent nature requiring anticoagulation, permanent pacing dependency of new onset)
- Major bleeding (chest tube blood loss at 18 h after surgery over 20 mL/kg) or need of massive red blood cell transfusion (5 or more units of packed red blood cells or an equivalent volume of washed red blood cells within intervention period)
- Infection compromising post-procedural rehabilitation
- AKI (postoperative creatinine at least two times compared to the preoperative level), renal replacement therapy

### Secondary outcome measures

- Total number of MAEs (several MAEs per patient to be counted)
- Incidence of major adverse cardiac event (MACE; cardiac death, acute myocardial injury, new onset acute heart failure, arrhythmia)
- Amount of each type of blood products transfused: a) red blood cell units, b) fresh frozen plasma units, c) platelet units
- Total fluid balance at the end of the intervention period
- Total measured blood loss (drainage)
- AKI development
- Days alive without mechanical ventilation in 90 days
- Days alive outside ICU in 90 days
- Days alive at home in 90 days
- 90-day mortality

### Adverse events and adverse reactions

Adverse events, serious adverse events (SAE), and suspected unexpected serious adverse reactions (SUSAR) are defined as described in the ICH-GCP. It is, however, recognized that cardiac surgery itself will result in typical aberrations in laboratory values, signs, and symptoms. These will not be classified as adverse events unless they are considered to be causally related to the study intervention. Events which are considered to be related directly to typical aberrations of cardiac surgery will not be classified as adverse events and are listed in Additional file 2.

### Data collection and patient follow-up

All relevant demographic and medical data, including outcome measures and adverse events, will be collected in electronic case report forms (CRF). In the operation theatre and ICU, patients will be monitored continuously according to the clinical routine, and all data will be extracted from the electronic patient data management system (Picis Clinical Solutions, version 8.2.13, Wakefield, MA, USA) using an information technology application tailored for the study. On the ward of the study site (Helsinki University Hospital), the study nurse will collect laboratory results manually. The study nurse will review continuous clinical medical notes during the hospital stay at the time of patient discharge from the study site (Helsinki University Hospital). The study nurse will screen all medical notes and laboratory results of all hospital visits during the 90-day follow-up period (Fig. 1). After the follow-up period, the patients will be interviewed per telephone.

### Data monitoring

HYKS-institute Ltd will undertake external monitoring of the validity of the trial conduct and data collection. At least five first patients will be monitored throughout. Thereafter, all patients will be monitored for eligibility (inclusion and exclusion criteria), consent, operation diagnosis, operation type, and primary outcome measures. In addition, 10% of the patients in a random manner will be monitored throughout. In addition to the above-mentioned parameters, this includes the following parameters: comorbidities, preoperative regular medication, and laboratory values during preoperative screening; administered intravenous fluids, blood products, and hemostatic drugs as well as diuresis and bleeding during the intervention period; core laboratory values throughout the study period; and adverse events and serious adverse events throughout the study period.

### Sample size analysis

In the literature, the incidence of MACE after cardiac surgery varies between 11 and 40%. The primary end-point in the present study, composite MAE, is broader than MACE and includes the latter. In a previous cohort study at the study site (Helsinki University Hospital, Meilahti hospital) the incidence of MAE was 30%. Thus, this incidence was used in the sample size analysis. Originally, it was estimated that 621 patients per group would be required to detect a 7.5%-unit absolute difference between the study groups in the primary outcome measure (proportion of patients with at least one MAE) with a two-sided significance level α = 0.05, and power of 80%. Accordingly, the original sample size was 1250 patients.

A predefined confirmatory analysis of incidence of MAE among the first 550 patients was performed for potential adaptation of the sample size in order to preserve the 80% power to detect a 7.5%-unit absolute difference between the study groups. The MAE incidence after 550 patients was 42%. In empirical analysis (without breaking the allocation code) the assumption of 50% and 42.5% MAE incidence in the two study groups turned out to be the “worst case scenario” (i.e., the highest needed patient number) with an estimate of 693 patients required for both patient groups. In accordance with the new power analysis, the sample size was increased by 2 × (693 − 1250/2) = 136 patients. Hence, the final adapted sample size will be 1250 + 136 = 1386. The Data and Safety Monitoring Board (DSMB) approved the extension of sample size and a decision was made before the interim analysis of 600 patients. Withdrawn patients or those who drop out, for whom the primary endpoint cannot be assessed, will be replaced.

### Statistical analyses

The primary efficacy endpoint will be the proportion of patients with at least one MAE in the study groups during the study period of 90 days. Conclusions from this study will be based on analyses conducted under the principle of intention-to-treat. For primary outcome, The Lan-DeMets spending function with O’Brien-Fleming type boundaries will be employed to preserve the overall two-sided type I error rate at the 0.05 significance level. The main conclusions of the study on primary outcome will be based on Fisher’s exact test between the treatment groups. The exact boundary values are *p* = 0.00132 in the interim analysis and *p* = 0.04868 in the final analysis.

For the primary outcome variable and for MACE and AKI incidence of the secondary outcomes, a time-to-event analysis will also be performed. The timing of MAEs is classified into four categories: 1) in the operating theatre, 2) in the intensive care unit, 3) during the primary stay at the ward of Meilahti hospital, and 4) after the primary stay at Meilahti hospital during the 90-day follow-up period.

For secondary outcomes, the proportions are compared with Fisher’s exact text and logistic regression models. For continuous variables, *t*-test and linear regression models will be used to evaluate differences between the treatment groups. All the analyses regarding the secondary outcomes will be conducted with a two-sided significance level of 0.05. No correction for the significance level will be done due to multiple testing.

Subgroup analyses will be conducted by preoperative glomerular filtration rate (GFR), EuroScore, and operation type. For GFR and EuroScore, the patients will be divided into two groups: those with a value higher and those with a value lower than the median value. For operation type, the patients will be divided into those operated on for aortic valve stenosis (including patient with other surgical corrections in addition to aortic valve stenosis) and those patients without correction of aortic valve stenosis.

The safety analysis is based on comparison of serious adverse events (SAE) between the study groups. Due to its nature, a MAE is always also a SAE. However, a MAE is not reported as a SAE in order to avoid double reporting. Consequently, the final number of SAEs is the sum of MAEs and the events reported as a SAE. Because a single patient may have more than one SAE, three different statistical analyses will be conducted for evaluation of study safety:

- The number of patients with at least one SAE using Fisher’s exact test
- The SAE number per a patient within those study patients with at least one SAE using *t*-test
- The SAE number per a patient within all study patients using *t*-test

### Interim analysis

One interim analysis will be conducted after 600 patients have been recruited and followed for 90 days. The principle of intention-to-treatment will be followed in all group comparisons of the interim analysis. The primary efficacy analysis (MAE) and safety analysis (MAE + SAE) will be conducted as described in the “Statistical analyses” section above.

The Data and Safety Monitoring Board will recommend pausing or stopping the trial if group difference in the primary outcome measure (proportion with at least one MAE) is found to be statistically significant at group sequential monitoring boundaries using Lan-DeMets spending functions. The exact boundary value for the interim analysis is *p* = 0.00132.

### Unblinding

The trial will be double-blind. For safety reasons, however, a premature unblinding of a specific patient may take place in the following situations:

- The investigator may request unblinding in case of an immediate safety hazard to the patient. Separate sealed envelopes for every allocation number will be available in this case.
- In case of a putative SUSAR, HUS Pharmacy, which will undertake randomization, will communicate directly with the Qualified Person Responsible for Pharmacovigilance of Sanquin Plasma Products B.V.

For interim analysis and after database lock at the end of the study, HUS-Pharmacy, which will generate the randomization and does not take part in patient care by any means, will separate the patients into two blinded treatment groups (marked by only “group 1” and “group 2”) for statistical analysis. The statistician who performs the analyses will be blinded for the treatment of these groups. The final unblinding will occur only after statistical analysis has been fully performed.

### Ethics approval, consent to participate, and ethical considerations

The operative ethics committee of Helsinki University Hospital approved the study protocol on 6 February 2017 (HUS/2917/2016). In addition, the Finnish Medicines Agency (Fimea, 136/ 2015) has approved the study protocol. Any modifications to the protocol will be immediately communicated to all responsible authorities. The trial will be performed according to the current version of the Helsinki Declaration (2013).

Eligible patients who undergo cardiac surgery at Meilahti Hospital (Helsinki University Hospital, Helsinki, Finland) will be recruited to the study. After obtaining informed consent, elective patients will be recruited during the perioperative screening visit for anesthesia. The patients scheduled for surgery during the index admission will be recruited as soon as the decision to perform surgery has been made and informed consent has been obtained.

Ringer acetate is used as conventional fluid therapy at the study site. In addition, albumin solutions are used after clinical consideration perioperatively. In a scientific literature review during the study design in 2013, a total of 25 RCTs with 1141 patients were found (Additional file 3). In priming fluid the maximum albumin dose was 80 g and the highest albumin concentration was 4%. The highest albumin dose for volume resuscitation was 254 g. The highest total albumin dose (including priming and volume resuscitation) was 267 g. No adverse events or transmitted infections were reported. In the present trial, the albumin dose in the priming fluid will be 60 g and the concentration 4%. The highest possible albumin dose for volume resuscitation will be 128 g, and thus the highest total albumin dose will be 188 g. According to the existing literature, the albumin doses administered in the present study are considered safe.

## Discussion

Optimal fluid therapy in adult cardiac surgery is not known. Patients receive large amounts of fluids during surgery, even though avoiding fluid overload and fluid extravasation may reduce complications [1–3]. Colloid use in cardiac surgery is preferred, but the data on the safety of HES solutions remain uncertain [10, 11, 26]. Thus, the assessment of the safety and efficacy of albumin solution in cardiac surgery is warranted.

The SAFE study is the only double-blind large scale trial on albumin use in fluid resuscitation thus far [27]. It compared the effect of albumin to saline on 28-day mortality in intensive care patients [27]. In terms of the clinical setting, there will be an essential difference between the SAFE study and the present study. In critically ill patients of the SAFE study, systemic inflammatory response and endothelial glycogalyx destruction had already begun before fluid resuscitation was initiated. This may have led to extravasation of the study fluid irrespective of its composition. In cardiac surgery glycogalyx is intact before CPB is initiated. Albumin in CPB priming prevents CPB-induced hypoalbuminemia and may reduce endothelial glycogalyx destruction and, thus, it may maintain microcirculatory function better [23, 24].

The theoretical basis of the present study is two-fold. First, by using albumin from the beginning of surgery and in CPB priming, i.e., before the onset of CPB, we hypothesize that albumin protects endothelial glycogalyx from destruction, preserves the microcirculation, and thus reduces end-organ dysfunction. Albumin may protect the glycocalyx also by reducing hypervolemia [19]. Second, albumin does not impair blood coagulation [15]. The primary outcome measure of the present study will be the composite end-point of MAEs, i.e., death, acute myocardial injury, acute heart failure, resternotomy, stroke, major arrhythmia, major bleeding, marked infection, or acute kidney injury. The primary outcome measure reflects both end-organ dysfunction and bleeding diathesis.

The present study will explore the effect and the safety of the 4% albumin in cardiac surgery with CPB compared to Ringer’s acetate. This study is the first large scale randomized, controlled, double-blind trial comparing albumin to crystalloid in cardiac surgery. No other such trial was registered in ClinicalTrials.gov as of July 31, 2019.

### Trial status

The first patient was enrolled on 21 March 2017 and the study is ongoing. At the time of submission the ALBICS trial has enrolled 1143 patients. The patient recruitment is expected to be completed in February 2020 and the follow-up in May 2020. The final protocol version is version 5, 3 September, 2018.

## Supplementary information

**Additional file 1.** SPIRIT checklist.

**Additional file 2.** Cardiac surgery-related aberrations in signs, symptoms, and laboratory values.

**Additional file 3.** References for literature review of safety of albumin solution in comparison with crystalloid in adult cardiac surgery.

**Additional file 4.** Ethics Committee approvals and Finnish Medical Agency (FIMEA) approvals.

## Data Availability

According to the prevailing confidentiality regulations even pseudonymized data cannot be placed on a public domain. Only the designated trial investigators will have access to the personal data of participants and to the final data set.

## Abbreviations

AKI: Acute kidney injury
cGMP: Current Good Manufacturing Practise
CK-MB: Creatinine kinase muscle/brain isoenzymes
CPB: Cardiopulmonary bypass
CRF: Case report form
ECMO: Extracorporeal membrane oxygenation
EDTA: Ethylenediaminetetraacetic acid
EF: Ejection fraction
EVA: Ethyl vinyl acetate
GFR: Glomerul filtration rate
HES: Hydroxyethyl starch
ICH-GCP: International Council for Harmonisation Good Clinical Practice
ICU: Intensive care unit
MACE: Major adverse cardiac event
MAE: Major adverse effect
MAP: Mean arterial pressure
PRBC: Packed red blood cell
RCT: Randomized clinical trial
S1P: Sphigosine-1-phosphate
SAE: Serious adverse event
SPIRIT: Standard Protocol Items: Recommendations for Interventional Trials
SUSAR: Suspected unexpected serious adverse reaction
