# Supplementary material for: Effect and safety of 4% albumin in the treatment of cardiac surgery patients: study protocol for the randomized, double-blind, clinical ALBICS (ALBumin In Cardiac Surgery) trial
Source: Trials. 2020 Feb 28;21:235. doi: 10.1186/s13063-020-4160-3 (PMC7048052; doi:10.1186/s13063-020-4160-3)
Supplement: Supplementary file 1 — Additional file 1. SPIRIT checklist. [file 13063_2020_4160_MOESM1_ESM.docx]

**Additional file 1. Albumin in cardiac surgery (ALBICS) Trial Standard Protocol Items: Recommendations for Interventional Trials (SPIRIT) protocol summary.**

| Section/item | Item No | Description | Addressed on page number |
| --- | --- | --- | --- |
| **Administrative information** | | |  |
| Title | 1 | Effect and safety of 4 % albumin in the treatment of cardiac surgery patients: study protocol for the randomized, double-blind, clinical ALBICS (ALBumin In Cardiac Surgery) trial | 1 |
| Trial registration | 2 | EudraCT number 2015-002556-27 and ClinicalTrials.gov number NCT02560519. | 3 |
| Protocol version | 3 | Version 5, 3 Sept 2018 | 3 |
| Funding | 4 | Sanquin Plasma Products B.V. (SPP), the Netherlands  VTR grant of Helsinki University Hospital | 24 |
| Roles and responsibilities | 5a | Authors: Hanna Vlasov, Tatu Juvonen, Seppo Hiippala, Raili Suojaranta, Markku Peltonen, Alexey Schramko, Kaapo Arvonen, Ulla-Stina Salminen, Ilona Kleine Budde, Tiina Eränen, Maxim Mazanikov, Mihkel Meinberg, Tommi Vähäsilta, Erika Wilkman, Ville Pettilä, Eero Pesonen. TJ, SH, RS, AS, KA, USS, EW, IKB, VP and EP are responsible for study design. HV and EP drafted the current manuscript. All authors reviewed and evaluated the manuscript. MP has responsibility for statistical plan. | 1, 24 |
|  | 5b | Primary sponsor: Eero Pesonen, Helsinki University Hospital, [eero.pesonen@hus.fi](mailto:eero.pesonen@hus.fi). | 24 |
|  | 5c | Sanquin Plasma Products B.V., the Netherlands will conduct pharmacovigilance activities and reporting to the competent authorities. Employees of SPP will co-author the scientific article of the trial that will be published in a medical journal. Reasonable comments made by SPP will be incorporated in the publication. However, in a case of a disagreement, SPP and Principal Investigator will mutually try to resolve the disagreement. | 24 |
|  | 5d | Data and safety monitoring board (prof. Anders Perner, Copenhagen, Denmark; prof. Markku Peltonen, Helsinki, Finland; prof. Jouko Jalonen, Turku, Finland) will interpret the results of the interim analysis of the first 600 patients. | 24 |
| Introduction |  |  |  |
| Background and rationale | 6a | In cardiac surgery with cardiopulmonary bypass (CPB), CPB priming with crystalloid solution causes marked hemodilution and fluid extravasation. Colloid solutions may reduce fluid overload because they have better volume expansion effect than crystalloids. The European Medicines Agency does not recommend the use of hydroxyethyl starch solutions (HES) due to harmful renal effect. | 2 |
|  |  | Albumin solution does not impair blood coagulation but the findings of kidney function are conflicting. On the other hand, albumin may reduce both destruction of endothelial glycocalyx and drop of platelet count during CPB. There are no large randomized, double-blind, clinical trials comparing albumin solution to crystalloid solution in cardiac surgery. |  |
|  | 6b | Control treatment: Ringer’s acetate solution (crystalloid) is the prevailing routine of the study site for CPB priming and perioperative intravenous volume replacement therapy.  Active treatment: Albumin solution based on study hypothesis. | 5, 6, 7 |
| Objectives | 7 | We hypothesize that CPB priming and intravenous volume replacement therapy with 4% albumin causes less major adverse events (MAE) than Ringer’s acetate solution in patients undergoing cardiac surgery with CPB. | 6 |
| Trial design | 8 | The study is a randomized, controlled, double-blind, clinical  single-center trial. | 7 |
| Methods: Participants, interventions, and outcomes | | |  |
| Study setting | 9 | Data will be collected in Helsinki University Hospital, Helsinki, Finland. | 7 |
| Eligibility criteria | 10 | Inclusion criteria   - age 18-90 years - scheduled for elective surgery or operated   during the index admission   - primary or repeat open heart surgery   procedures, either independently or in  combinations   - - - coronary artery bypass grafting     - aortic valve replacement/repair     - mitral valve replacement/repair     - tricuspid valve replacement/repair     - the maze procedure or its modifications     - aortic root or ascending aorta surgery when no hypothermic circulatory arrest is required   Exclusion criteria   - immediate emergency surgery (i.e. no time for   recruitment)   - correction of a congenital cardiac defect | 7,8 |
|  |  | - preoperative infection compromising   post-procedural rehabilitation   - hemophilia A, hemophilia B - patient refusal of the use of blood products and   derivatives of blood products   - end-stage kidney disease (estimated   glomerular filtration rate (GFR) <20 mL/min –  based on serum/plasma creatinine)   - preoperative heart failure/low output syndrome defined as preoperative inotropic support, preoperative mechanical assistance of breathing, preoperative extracorporeal membrane oxygenation (ECMO) support, preoperative intra-aortic balloon pump, preoperative mechanical assistance of left ventricle, preoperative ejection fraction (EF)   < 20% (intraoperative transesophageal echocardiography not included), some other comparable preoperative conditions; the condition has to be ongoing   - ticagrelol, prasugrel or clopidogrel treatment   within 2 days   - apixaban or rivaroxaban treatment within 2   days or dabigatran treatment within 3 days |  |
|  |  |  |  |
| Interventions | 11a | In the first phase of the trial, the study solution will be used  for priming of the CPB circuit. In the second phase of the  trial, during surgery and during the first 24 hours in the  intensive care unit (ICU), study solution will be used as  volume replacement therapy up to 3200 mL. If more than  3200 mL of volume replacement will be needed during the  second study phase, Ringer’s acetate solution will be used thereafter. The volume replacement therapy will not be  protocolled and will be based on the clinical decision.  ***Albumin group***   - The first phase: The CPB circuit will be initially   primed with 1200 mL of Ringer’s acetate solution in an unblinded manner. Thereafter, in a blinded manner,  300 mL of 20% (200 g/L) albumin solution  (Albuman® 200 g/L, Sanquin Plasma Products BV, the Netherlands) will be added, resulting in the final  albumin concentration of 4% in the priming fluid.   - The second phase: 4% (40 g/L) albumin solution (Albuman®, Sanquin Plasma Products BV, the Netherlands) in a blinded manner. | 8, 9, 10 |
|  |  |  |  |
|  |  |  |  |
|  |  | ***Ringer group***   - The first phase: The CPB circuit will be initially primed   with 1200 mL of Ringer’s acetate solution in an  unblinded manner. Thereafter, in a blinded manner,  300 mL of Ringer’s acetate solution (RingerAcetat  Baxter Viaflo®, Baxter, Finland) will be added,  resulting in pure Ringer’s acetate solution as the  priming fluid.   - The second phase: Ringer’s acetate solution   (RingerAcetat Baxter Viaflo®, Baxter, Finland) in a  blinded manner.  The intervention period will end after the first 24 hours of ICU  stay or when the patient leaves the ICU if the latter will occur within the first postoperative 24 hours. After the intervention period, fluids will be administered according to the local  clinical practice. The use of blood products, excluding albumin, will  not be protocolled. For physiological basic need of fluid,  Ringer’s acetate solution will be given throughout the  intervention period as a background infusion of 0.5 mL/kg/h, rounded to the nearest multiple of 10 mL. Apart from the  study intervention, patients will be treated according to the  clinical practice of the hospital. |  |
|  | 11b | The trial will be double-blind. For safety reasons, however, a premature unblinding of a specific patient may take place in the following situations:   - The investigator may request unblinding in a case of an immediate safety hazard to the patient. - In a case of a putative suspected unexpected serious adverse reaction | 19 |
|  | 11c | Not relevant. The intervention consists of CPB priming and perioperative intravenous fluid therapy in the operation room and intensive care unit. |  |
|  | 11d | The use of albumin is prohibited during the intervention time. | 9 |
| Outcomes | 12 | ***Primary outcome measure***  The primary efficacy endpoint will be the proportion of  patients with at least one major adverse event (MAE) during  the study period of 90 days. The composite end-point of  MAE was adopted with small modifications from a previous publication.^26^ | 14, 15, 16 |
|  |  | MAE composition   - all-cause death - acute myocardial injury (defined as an increase   in creatinine kinase muscle/brain isoenzymes  (CK-MB) at least 10 times as high as the upper normal limit of the local laboratory, i.e. 70 µg/l)   - new onset of acute heart failure   (e.g. pulmonary edema, cardiogenic shock), or  low output syndrome requiring intravenous  inotropic agents and/or intra-aortic balloon  pump support, ECMO support or other  comparable condition   - resternotomy, subxiphoidal pericardial   drainage or other comparable postoperative procedure   - stroke - major arrhythmia (ventricular fibrillation   off-CPB, ventricular tachycardia off-CPB,  new onset atrial fibrillation of permanent nature requiring anticoagulation, permanent pacing dependency of new onset) |  |
|  |  | - major bleeding (chest tube blood loss at 18   hours after surgery over 20 mL/kg) or need of  massive red blood cell transfusion (5 or more  units of packed red blood cells or an  equivalent volume of washed red blood cells  within intervention period   - infection compromising post-procedural   rehabilitation   - AKI (postoperative creatinine at least 2 times compared to the preoperative level), renal replacement therapy   ***Secondary outcome measures***   - total number of MAEs (several MAEs per   patient to be counted)   - incidence of major adverse cardiac event   (MACE: cardiac death, acute myocardial injury,  new onset of acute heart failure, arrhythmia)   - number of each type of blood products   transfused: a) red blood cells units, b) fresh  frozen plasma units, c) platelet units   - total fluid balance at the end of the intervention   period |  |
|  |  | - total measured blood loss (drainage) - AKI development - days alive without mechanical ventilation in   90 days   - days alive outside ICU in 90 days - days alive at home in 90 days - 90-day mortality |  |
| Participant timeline | 13 | Eligible patients who undergo cardiac surgery at Meilahti Hospital (Helsinki University Hospital, Helsinki, Finland) will be recruited to the study. After obtaining informed consent, elective patients will be recruited during the perioperative screening visit for anesthesia. See Figure 1 on page 32 for schedule of procedures. The patients scheduled for surgery during the index admission will be recruited as soon as the decision of surgery has been made and the informed consent has been obtained. | 27, 32 |
| Sample size | 14 | In a previous cohort at the study site (Helsinki University Hospital) the incidence of MAE was 30%. This incidence was used in the original power analysis. Originally, it was estimated that 621 patients per group will be required to detect a 7.5%-unit absolute difference between the study groups in the primary outcome measure with a two-sided significance level α=0.05, and the power of 80%. | 17, 18 |
|  |  | Accordingly, the original sample size was fixed at 1250 patients. A predefined confirmatory analysis of incidence of MAE among the first 550 patients was performed for potential adaptation of the sample size in order to preserve the power of 80% to detect a 7.5%-unit absolute difference between the study groups. The MAE incidence after 550 patients was 42%. In empirical analysis (without breaking the allocation code) the assumption of 50% and 42.5% MAE incidences in the two study groups turned out to be the “worst case scenario” with an estimate of 693 patients required for both patient groups. In accordance with the new power analysis, the sample size was increased to1386 patients. |  |
| Recruitment | 15 | Eligible patients who undergo cardiac surgery at Meilahti  Hospital (Helsinki University Hospital, Finland) will be  recruited and asked for a written informed consent. | 21 |
| **Methods:** |  | **Assignment of interventions (for controlled trials)** |  |
| Allocation: | 16a | Randomization will be done every other week at HUS Pharmacy (the hospital pharmacy of Helsinki University Hospital) with an online software ([www.randomization.com](http://www.randomization.com)) in conjunction with the production of the blinded study solution bags. The personnel of HUS Pharmacy will not take | 11, 12 |
| Sequence generation, allocation concealment mechanism and implement-tation |  | part in patient care. The entire study group and all personnel taking care of the patients will be kept blinded to randomization. HUS Pharmacy will deliver a study solution bag set for each patient. For each set, HUS Pharmacy will give a unique consecutive allocation number. Randomization of consecutive allocation numbers into the two study arms will be performed in blocks before preparation of each lot of the study solution bag sets. The actual patient randomization will occur preoperatively when the next available study solution bag set for the subsequent study patient will be taken into use. Varying block sizes will be used. The block sizes will be chosen to match the estimated patient recruitment rate within the shelf life of the study solution bags. Preferentially, the block sizes will be 24 patients and 30 patients, alternating every other preparation lot. Other block sizes may be used if needed. In the blocks, patients will be randomized to the two treatment groups in the ratio of 1:1, except for the last few blocks of the study. |  |
| Blinding (masking) | 17a | The personnel of HUS Pharmacy, which will conduct randomization, will not take part in patient care. The entire study group and the personnel taking care of the patients will be kept blinded to randomization. The study is double-blinded. Albumin solution is yellow and slightly viscous | 12, 13, 14 |
|  |  | whereas Ringer’s acetate is colourless. All study solutions will be delivered in non-transparent study solution bags. Infusion tubings will be coloured and drop chambers are covered with non-transparent tape. Study solutions will be administered with infusion pumps. In CPB priming, the tube, which is used for filling the CPB reservoir, will be covered with non-transparent tape. The initial priming of Ringer’s solution will be made coloured with patient blood before adding of study solution. |  |
|  | 17b | The trial will be double-blind. For safety reasons, however, premature unblinding of a specific patient may take place in the following situations:   - The investigator may request unblinding in a case of an immediate safety hazard to the patient. - In a case of a putative suspected unexpected serious adverse reactions. | 20 |
| **Methods: Data collection, management, and analysis** | | |  |
| Data collection methods | 18a | All relevant demographic and medical data, including outcome measures and adverse events will be collected in electronic case report forms (CRF). In the operation theatre and ICU, patients will be monitored continuously according to the clinical routine, and all data will be extracted from the electronic patients data management system (Picis Clinical Solutions, version 8.2.13, Wakefield, MA, USA) using an | 16 |
|  |  | information technology application tailored for the study. On the ward of study site (Helsinki University Hospital), the study nurse will collect laboratory results manually. The study nurse will review continuous clinical medical notes during the hospital stay at the time of patient discharge from the study site (Helsinki University Hospital). |  |
|  | 18b | The study nurse will screen all medical notes and laboratory results of all hospital visits during the 90-day follow-up period. After the follow-up period, the patients will be interviewed per telephone. The patient (or in the case of proxy-consent, the person who has given the consent) has the right to withdraw consent for participation to the study at any time. The patient/proxy asking for withdrawal from the study will be separately asked for permission to continue data registration, or at least for the use of registered data and the primary endpoint. Withdrawn or dropped out patients of whom primary endpoint cannot be assessed will be replaced. Patients who will be unblinded due to immediate safety hazard will be included in the statistical analysis if the primary endpoint can be assessed. For the statistician these patients will be kept blinded. | 15, 20 |
| Data management | 19 | A professional service provider will conduct data management. All electronically extracted data will be saved, along with back-ups, on the server of the service provider. The study data is secured with a role-based access right management. The architecture of the application has two layers: One server for running the application and another server for storing the data. Access to the latter server requires a VPN connection with a strong authentication. In addition, all communication with either of the servers requires a separate and strongly verified user identification. The server environment fulfils the KATAKRI criteria.  Only the study group, the personnel of the Department Clinical Operations of Sanquin and the external monitor (HYKS-Institute Ltd) have access to the study data.  In the study data, the patients will be pseudonymized and presented with their allocation numbers. The personal identification data of the patients with the corresponding trial allocation numbers will be stored in a separate file requiring strongly verified user identification. | 16 |
| Statistical methods | 20a | Intention-to-treat analysis  The primary analysis (efficacy) will be group comparison of proportions with composite MAE in two treatment groups and the statistical significance will be evaluated with Fisher’s exact test. | 18, 19 |
|  |  | For the primary outcome variable and for MACE and AKI incidence of the secondary outcomes, also a time-to-event analysis will be performed. The safety analysis will be based on comparison of serious adverse events (SAE) between the study groups.   - The number of patients with at least one SAE using Fisher’s exact test. - The SAE number per a patient within those study patients with at least one SAE using t-test.   The SAE number per a patient within all study patients using t-test. |  |
|  | 20b | Subgroup analyses will be conducted by preoperative GFR, EuroScore and operation type (patients with vs. without aortic stenosis). |  |
| **Methods: Monitoring** | | |  |
| Data monitoring | 21a | HYKS-institute Ltd will perform the monitoring of the trial to ensure compliance with the study protocol and with good clinical practice (GCP), and that data is accurate, complete, and verifiable. At least 5 first patients will be monitored throughout. Thereafter all patients will be monitored for eligibility (inclusion and exclusion criteria), consent, operation diagnosis, operation type, and primary outcome measures. In addition, 10% of the patient in a random manner will be monitored throughout. | 17 |
|  | 21b | Interim analysis will be conducted after 600 patients have been recruited and followed for 90 days. Data and safety monitoring board (prof. Anders Perner, Copenhagen, Denmark; prof. Markku Peltonen, Helsinki, Finland; prof. Jouko Jalonen Turku, Finland) will interpret the results of the interim analysis. | 18 |
| Harms | 22 | Adverse events, serious adverse events (SAE) and suspected unexpected serious adverse reactions will be defined in a standard manner according to the International Council for Harmonisation Good Clinical Practice guideline (ICH-GCP). Events that are listed in the Additional file 2 are considered as typical aberrations of laboratory values, signs and symptoms to be related directly to cardiac surgery and will not be classified as adverse events. SAEs will be assessed for seriousness and potential causal relationship by the Investigator and Sanquin. | 16,  44-47 |
| Auditing | 23 | No audit will be conducted. |  |
| Ethics and dissemination | | |  |
| Research ethics approval | 24 | The local ethics Committee and Finnish Medical Agency (FIMEA) have approved the study protocol. | 21 |
| Protocol amendments | 25 | The first protocol revision concerning extended exclusion criteria and reformulated definitions of major adverse events was accepted on June 14, 2017.  The second protocol revision concerning extended patient recruitment was accepted on January 18, 2019. | 52-59 |
| Consent or assent | 26a | Eligible patients who undergo cardiac surgery at Meilahti  Hospital (Helsinki University Hospital, Finland) will be  recruited. Elective patient will be asked for written informed consent during the perioperative screening visit.  Patients scheduled for surgery during the index admission  will be asked for consent as soon as the decision of surgery  has been made. | 21 |
|  | 26b | Not relevant. |  |
| Confidentiality | 27 | All relevant demographic and medical data, including outcome measures and adverse events will be collected in electronic case report forms (CRF). All data will be pseudonymized. The study data will be secured with a role-based access-right management with strong authentication. | 16 |
| Declaration of interests | 28 | Main financing of the trial will be provided by Sanquin Plasma Products B.V.. Still, Principal Investigator will own the Intellectual Property Rights and Now How arising from clinical | 25 |
|  |  | trial not relating directly to Sanquin’s Albuman product. Sanquin will own the Intellectual Property Rights and Now How arising from clinical trial relating directly to Sanquin’s Albuman product. In a case of a disagreement in the content of the scientific publication of the trial, Sanquin and Principal Investigator will mutually try to resolve the disagreement. |  |
| Access to data | 29 | Only the designated trial investigators will have access to the personal data of participants and to the final data set. | 16 |
| Ancillary and post-trial care | 30 | The study patients are covered by the insurance of the hospital. Otherwise, the patients will not receive any compensation through participation in the study. |  |
| Dissemination policy | 31a | According to the prevailing confidentiality regulations even pseudonymized data cannot be placed on a public domain. | 16 |
|  | 31b | Not relevant. |  |
|  | 31c | Not relevant. |  |
| Appendices |  |  |  |
| Informed consent materials | 32 | Informed consent and information leaflet are provided as additional files, see below. |  |
| Biological specimens | 33 | Plasma samples for biochemical analyses will be collected at four timepoints. |  |

**Additional file to the SPIRIT checklist: ALBICS-trial information leaflet to the patient.**

For the patient

**Patient information regarding a fluid therapy trial for patients undergoing cardiac surgery**

**ALBICS - Albumin in cardiac surgery (Eudra-CT 2015-002556-27)**

**Background**

You are going to have cardiac surgery, which involves the use of a cardiopulmonary bypass machine. The tubing of the cardiopulmonary bypass machine needs to be filled with fluid before the machine can be used. This can be done with either albumin solution or a so-called Ringer’s solution. Both albumin solution and Ringer’s solution are commonly used in intravenous fluid therapy and in connection with cardiac surgery. Patients undergoing cardiac surgery will receive post-operative intravenous fluid therapy at the intensive care unit based on their individually evaluated needs. Either albumin solution or Ringer’s solution can be used for this treatment. There is no reliable research data on the superiority of either of these therapies. However, previous trials have shown that albumin plays an important part in maintaining the structural balance of the walls of blood vessels. By using albumin solution, we might also be able to reduce the need for post-operative fluid therapy, which could have a beneficial effect on recovery from surgery.

In this trial, Ringer’s solution and albumin solution will be compared with each other in connection with cardiac surgery. Ringer’s solution and albumin solution are two of the most commonly used infusion solutions in cardiac surgery, both in Meilahti Hospital and internationally. Even if you choose not to participate in this trial, it is possible that you will receive one or both of these solutions in line with standard treatment practice. Therefore, in this trial we will compare fluids that are already being routinely used in cardiac surgery, both in Meilahti Hospital and internationally.

We are asking for your consent to participate in an ongoing clinical trial comparing the use of albumin solution (trial group) and Ringer’s solution (control group) in a cardiopulmonary bypass machine and in intravenous fluid therapy in patients undergoing cardiac surgery. Please read this information carefully. You can also discuss the trial with the trial doctor. If you consent to participate in this trial, we will ask you to sign the patient’s consent form. Participation in this trial is completely voluntary.

**Purpose of the trial**

The purpose of this trial is to compare two different fluids, 4% albumin solution and Ringer’s solution, in a cardiopulmonary bypass machine and in post-operative fluid therapy given at an intensive care unit.

**Practical implementation**

During your cardiac surgery, the tubing of the cardiopulmonary bypass machine will be filled with either 4% albumin solution or Ringer’s solution. You will receive the same fluid as intravenous fluid therapy at the intensive care unit for the first 24 hours. After the first 24 hours, intravenous fluid therapy will be given in line with the hospital’s standard practice. Any other treatment will be given in line with the standard approved treatment practices.

During the trial, four blood samples will be collected from you. Two of them will be taken in the operating room and two at the intensive care unit after the surgery. The samples will be collected via the arterial cannula inserted in connection with the surgery. No extra needle sticks are needed because of these blood samples. The blood samples will be tested for albumin levels, inflammatory reactions (IL-6, IL-8, IL-10), white blood cell activation (myeloperoxidase), blood clotting (coagulation factors, platelets, F1+2), fibrinolysis (tPA, PAI-1), and glycocalyx degradation (sphingosine-1-phosphate, syndecan-1, heparin sulphate). Other markers related to these can also be analysed if necessary. The samples will be kept in the trial team’s deep freezer in a lockable facility, and they will be destroyed once all the laboratory analyses specified in the trial protocol have been performed.

**Benefits of the trial**

You may benefit from participating in this trial because the fluid therapy you receive will be given in line with standardised treatment practice. This trial will produce more information about the effects of albumin in patients undergoing cardiac surgery and help develop the treatment given to patients.

**Possible risks of the trial**

Both 4% albumin solution and Ringer’s solution have been used in fluid therapy for decades, and they are routinely used in Meilahti Hospital and internationally. Albumin solution is produced by isolating albumin from blood plasma, which means that the use of albumin solution is theoretically associated with the risk of contracting a bloodborne disease. However, no such infections have been observed in large, international registration trials conducted with tens of thousands of patients, and albumin solution is generally considered to be a very safe infusion solution.

**Who can participate**

Patients undergoing cardiac surgery can participate in this trial.

**Who cannot participate**

Patients undergoing extremely urgent emergency surgery and patients who have been diagnosed with renal insufficiency cannot participate in this trial. The doctor in charge of the trial decides who will be enrolled into the trial.

**Withdrawing from the trial**

You can discontinue your participation in the trial at any time without giving a reason. Withdrawing from the trial will not have any effect on the treatment you receive or your patient-doctor relationship. You will receive the best possible care also after withdrawing from the trial.

The trial doctor can also discontinue your participation in the trial at his/her discretion.

**Confidentiality, data processing and storage**

Data and test results collected from you will be handled confidentially in the manner required by the Personal Data Act. Members of the trial team (anaesthesiologists and cardiac surgeons) and staff members assisting the trial team will handle the trial data. The trial data pre-specified in the trial protocol will be collected into the trial database from clinical data systems used for the implementation of your treatment. This will be done mostly automatically by means of information technology, and manually to a very modest degree. When the results are analysed and reported, individual patients cannot be identified from them. In addition to the investigators, trial data may be disclosed to the Finnish Medicines Agency Fimea (the regulatory authority in Finland), the trial’s independent monitoring group (consists of impartial doctors), Sanquin Plasma Products B.V. (responsible for recording data on drug safety and reporting them to the authorities), and the drug regulatory and safety authorities in the Netherlands. All these parties are bound by the same obligations of secrecy and confidentiality as the trial doctors. Trial data will not be disclosed to countries outside the EU/EEA. The data obtained in this clinical trial will be stored for a period of 15 years.

You can ask at any point if we are storing data about you, on what grounds, where the data were obtained from, and to whom your samples or data have been disclosed. You have the right to receive the data free of charge and within a reasonable period of time. The data will be submitted to you in writing, electronically or orally, and at any rate in a manner that allows reliable confirmation of your identity. You also have the right to request correction or supplementation of your data, if they are incorrect, incomplete or inaccurate.

In matters relating to data protection, you should primarily contact the persons in charge of the trial, whose contact details can be found at the end of the information letter you received with the consent form.

You can exercise your rights under the General Data Protection Regulation by using free-form notifications, but we recommend that you use the forms specifically prepared by HUS for this purpose, available on the HUS website:

[http://www.hus.fi/potilaalle/potilaan oikeudet/terveystieteellinen%20tutkimus/Sivut/default.aspx](http://www.hus.fi/potilaalle/potilaan_oikeudet/terveystieteellinen%20tutkimus/Sivut/default.aspx). You also have the right to file a complaint with the authority responsible for data protection in Finland, or the Data Protection Ombudsman (Office of the Data Protection Ombudsman, Ratapihantie 9, PO Box 800, 00521 Helsinki, tel. +358 (0)29 566 6700, e-mail: [tietosuoja@om.fi](mailto:tietosuoja@om.fi)).

Data controller for the trial: Hospital District of Helsinki and Uusimaa

Data controller for codes: Hospital District of Helsinki and Uusimaa, Department of Anaesthesiology, Intensive Care and Pain Medicine

**Trial financier**

Sanquin Plasma Products B.V. is primarily responsible for financing the trial. This company is owned by Sanquin Blood Supply Foundation, which is responsible for activities relating to blood products in the Netherlands and also manufactures blood and plasma products. The doctors responsible for carrying out the trial are not affiliated with the financier.

Legal grounds of the trial

This trial and the related processing of personal information are based on the following regulations: EU General Data Protection Regulation 2016/679, Article 6, paragraphs 1a, 1b, 1c and 1e, and Article 9, paragraphs 3a, 3g, 3i and 3j; Medical Research Act. In addition, an Ethics Committee has issued a favourable opinion on the trial.

Regulations applicable to this trial and the processing of personal information are also provided in the Health Care Act (1326/2010), the Act on the Status and Rights of Patients (785/1992), the Act on Health Care Professionals (559/1994), the Act on the Openness of Government Activities (621/1999), the Personal Data Act (523/1999) and the Archives Act (831/1994), taking into consideration, however, that some of the regulations given in the EU General Data Protection Regulation overrule national legislation.

**Insurance**

Patients participating in the trial are covered by the treating hospital’s insurance for the duration of the trial.

**Trial results**

Once the trial has been completed, the results will be published in international medical publications.

**Contact information**

The investigators in charge of the trial can provide you with more information about the trial if needed.

Eero Pesonen, Associate Professor, tel. +358 (0)50 4271784

Alexey Schramko, D.Med.Sc., tel. +358 (0)50 4270610

Kaapo Arvonen, Lic.Med., tel. +358 (0)50 4286276

**Additional file to the SPIRIT checklist. Consent for ALBICS-trial**

**CONSENT TO PARTICIPATE IN THE CLINICAL TRIAL**

I have been asked to participate in a trial comparing 4% albumin solution and Ringer’s solution in patients undergoing cardiac surgery.

I have received, read and understood the information leaflet concerning the trial. From this leaflet I have received an adequate clarification regarding the trial (ALBICS, albumin in cardiac surgery, Eudra-CT 2015-002556-27) and the collection, processing and disclosure of data in conjunction with the trial. The contents of the information leaflet have been explained to me also orally and I have received a sufficient answer to all my questions relating to the trial.

The information was given by , / / 20 .

I have had sufficient time to consider my participation in the trial.

I have been told where the information concerning me will be acquired from. I give consent to gathering information about me that is essential for the trial in the trial data file of Helsinki University Hospital. When the clinical trial necessitates, health care facilities which have my patient information may be asked for it. For this purpose, the doctor may record my personal identity code and use it to collect information.

All the information collected about me during the trial will be confidential. The information shall be coded in such a manner that exploration of identity is not possible later on without an identification code. The code is stored in the locked files of the doctor conducting the trial or Helsinki University Hospital.

The regulatory authorities responsible for pharmaceuticals and their safety (in Finland the Finnish Medicines Agency Fimea), have the right to conduct trial data verification and ensure the appropriate conduct of the trial. This is done by comparing the trial information to my original medical records and information regarding my state of health. I give consent to conducting data verification also to the representatives of Helsinki University Hospital, the independent institutional review board, Sanquin Plasma Products B.V. (the Netherlands) and the authorities responsible for drug regulation and safety in the Netherlands. In this case the information shall be verified at the responsibility and under the supervision of the doctor conducting the trial. All of the above mentioned instances are required to keep this information confidential. Trial data will not be disclosed to countries outside the EU/EEA.

I understand that my participation in this trial is completely voluntary. I have the right at any time during the trial to discontinue my participation in the trial or withdraw my consent without giving a reason. My refusal to participate in the trial, discontinuing my participation or withdrawing my consent will not affect my subsequent care. I am aware that information collected before I discontinue my participation or withdraw my consent will be used as part of the trial data. This information is of key importance in the safety assessment of the drug.

**With my signature I verify my participation in this trial and consent to being a voluntary trial subject.**

| Signature |  | Date |
| --- | --- | --- |
| Name in print |  | Date of birth |
| Address |  |  |
| **Consent received** |  |  |
| Signature of the trial doctor |  | Date |
| Name in print |  |  |

**The original signed subject consent and a copy of the information leaflet will remain in the files of the doctor conducting the trial. The subject information leaflet and a copy of the signed consent will be given to the trial subject.**
