# Supplementary material for: Effect and safety of 4% albumin in the treatment of cardiac surgery patients: study protocol for the randomized, double-blind, clinical ALBICS (ALBumin In Cardiac Surgery) trial
Source: Trials. 2020 Feb 28;21:235. doi: 10.1186/s13063-020-4160-3 (PMC7048052; doi:10.1186/s13063-020-4160-3)
Supplement: Supplementary file 2 — Additional file 2. Cardiac surgery-related aberrations in signs, symptoms, and laboratory values. [file 13063_2020_4160_MOESM2_ESM.docx]

Additional file 2: Cardiac surgery related aberrations in signs, symptoms and laboratory values.

It is recognized that cardiac surgery itself will result in typical aberrations in laboratory values, signs and symptoms. These are not classified as adverse events unless they are considered to be causally related to the study intervention. In other words, events which are considered to relate directly to cardiac surgery are not classified as adverse events. The following events are not adverse events:

Blood pressure

- mean arterial pressure (MAP) 50-140 mmHg (outside CPB)
- MAP 40-140 mmHg (during CPB)

Hypotension

- MAP lower than 50 mmHg, if duration less than 180 s (outside CPB)
- MAP lower than 35 mmHg, if duration less than 180 s (during CPB, deliberate hypotension during aortic cannulation and de-cannulation not included)

Hypertension

- MAP higher than 140 mmHg, if duration less than 180 s (outside CPB)
- MAP higher than 100 mmHg, if duration less than 180 s (during CPB)

Use of vasoactive drugs

- Norepinephrine (bolus + infusion, average)
- Dose less than 0.2 µg/kg^.^min
- Epinephrine (bolus + infusion, average)
  - Dose less than 0.05 µg/kg^.^min
- Phenylephrine (bolus)
- Ephedrine (bolus)
- Milrinone (bolus + infusion, average)
- Dose less than 0.5 µg/kg^.^min
- Dobutamine (bolus + infusion, average)
- Dose less than 10 µg/kg^.^min

Arrhythmias

- Atrial fibrillation
- Supraventricular tachycardia
- Nodal tachycardia
- Supraventricular extrasystolia
- Ventricular extrasystolia
- Ventricular tachycardia (during CPB)
- Ventricular fibrillation (during CPB)
- Asystole or any bradycardia

Conduction disturbances

- Sinoatrial block
- Atrioventricular block
- Left bundle branch block
- Right bundle branch block
- Left posterior hemiblock
- Left anterior hemiblock

ECG changes

- ST-segment elevation
- ST-segment depression
- QRS disconfiguration
- R-wave depression

Need for cardiac pacing

- Atrioventricular pacing
- Ventricular pacing
- Atrial pacing
- Pacing dependency

Deviation from normothermia

- Core temperature more than 28 ^o^C
- Core temperature less than 40 ^o^C

Anemia

- Hb more than 60 g/l (during CBP)
- Hb more than 70 g/l (outside CBP)

Blood product transfusion

- Red blood cells
- Fresh frozen plasma
- Platelet concentrates

Use of plasma products

- Prothrombin complex concentrate
- Fibrinogen

Deviations of laboratory parameters

- Leukocytes count more than 2^*^10^9^/l
- Platelet count more than 50^*^10^9^/l
- Any increase of CRP
- Tromboplastin time more than 25% (without warfarin)
- Plasma potassium concentration 2.5-7 mmol/l
- Plasma sodium concentration 125-155 mmol/l
- Increase in bilirubin less than 50% (compared to preoperative level)
- Hypo- or hypermagnesiemia
- Hypo- or hyperphosphatemia
- Hypo- or hypercalsiemia
- Hypo- or hyperchloremia
- pH 7.25-7.60
- pO2 over 7.5 kPa
- pCO2 3-8 kPa
- Any base excess
- Plasma lactate concentration under 5.0 mmol/l
