# Supplementary material for: Effect and safety of 4% albumin in the treatment of cardiac surgery patients: study protocol for the randomized, double-blind, clinical ALBICS (ALBumin In Cardiac Surgery) trial
Source: Trials. 2020 Feb 28;21:235. doi: 10.1186/s13063-020-4160-3 (PMC7048052; doi:10.1186/s13063-020-4160-3)
Supplement: Supplementary file 3 — Additional file 3. References for literature review of safety of albumin solution in comparison with crystalloid in adult cardiac surgery. [file 13063_2020_4160_MOESM3_ESM.docx]

**Additional file 3. References for literature review of safety of albumin solution in comparison with crystalloid in adult cardiac surgery.**

1. Hallowell P, Bland JH, Dalton BC, Erdmann AJ, Lappas DG, Laver MB et al. The Effect of Hemodilution with Albumin or Ringer's Lactate on Water Balance and Blood Use in Open-Heart Surgery. Ann Thorac Surg. 1978;25:22-29.
2. Öhqvist G, Settergren G, Bergström K, Lundberg S. Plasma colloid osmotic pressure during open-heart surgery using non-colloid or colloid priming solution in the extracorporeal circuit. Scand J Thorac Cardiovasc Surg. 1981;15:251-5.
3. Öhqvist G, Settergren G, Lundberg S. Pulmonary Oxygenation, Central Haemodynamics and Glomerular Filtration Following Cardiopulmonary Bypass with Colloid or Non-Colloid Priming Solution. Scand J Thorac Cardiovasc Surg.1981;15:257-62.
4. Gallagher JD, Moore RA, Kerns D, Jose AB, Botros SB, Flicker S, et al. Effects of colloid or crystalloid administration on pulmonary extravascular water in the postoperative period after coronary artery bypass grafting. Anesth Analg. 1985;64:753-8.

# Sade RM, Stroud MR, Crawford FA Jr, Kratz JM, Dearing JP, Bartles DM. A prospective randomized study of hydroxyethyl starch, albumin, and lactated Ringer's solution as priming fluid for cardiopulmonary bypass. J Thorac Cardiovasc Surg. 1985;89;713-22.

1. Marelli D, Paul A, Samson R, Edgell D, Angood P, Chiu RC. Does the addition of albumin to the prime solution in cardiopulmonary bypass affect clinical outcome? J Thorac Cardiovasc Surg;1989;98:751-6.

# McGrath LB, Gonzalez-Lavin L, Neary MJ. Comparison of dextran 40 with albumin and Ringer's lactate as components of perfusion prime for cardiopulmonary bypass in patients undergoing myocardial revascularization. Perfusion. 1989;4:41-9.

# Bonser RS, Dave JR, Davies ET, John L, Taylor P, Gaya H, et al. Reduction of complement activation during bypass by prime manipulation. Ann Thorac Surg 1990;49:279-83.

1. Hoeft A, Korb H, Mehlhorn U, Stephan H, Sonntag H. Priming of cardiopulmonary bypass with human albumin or ringer lactate: Effect on colloid osmotic pressure and extravascular lung water. Br J Anaest. 1991;66:73-80.
2. London MJ, Franks M, Verrier ED, Merrick SH, Levin J, Mangano DT. The safety and efficacy of ten percent pentastarch as a cardiopulmonary bypass priming solution. A randomized clinical trial. J Thorac Cardiovasc Surg. 1992;104:284-96.

# Jenkins IR, Curtis AP. The combination of mannitol and albumin in the priming solution reduces positive intraoperative fluid balance during cardiopulmonary bypass. Perfusion. 1995;10:301-5.

# Scott DA, Hore PJ, Cannata J, Masson K, Treagus B, Mullaly J. A comparison of albumin, polygeline and crystalloid priming solutions for cardiopulmonary bypass in patients having coronary artery bypass graft surgery. Perfusion. 1995;10:415-24

# Tollofsrud S, Svennevig JL, Breivik H, Kongsgaard U, Ozer M, Hysing E, et al. Fluid balance and pulmonary functions during and after coronary artery bypass surgery: Ringer's acetate compared with dextran, polygeline, or albumin. Acta Anesthesiol Scand. 1995;39:671-7.

# Svennevig JL, Tollofsrud S, Kongsgaard U, Noddeland H, Mohr B, Ozer M, et al. Complement activation during and after open-heart surgery is only marginally affected by the choice of fluid for volume replacement. Perfusion. 1996;11: 326-32.

# Buhre W, Hoeft A, Schorn B, Weyland A, Scholz M, Sonntag H. Acute affect of mitral calve replacement on extravascular lung water in patients receiving colloid or crystalloid priming of cardiopulmonary bypass. Br J Anaesth. 1997;79:311-6.

1. Saxena N, Chauhan S, Ramesh G. A comparison of Hetastarch, albumin and Ringer lactate for volume replacement in coronary artery bypass surgery. J Anaesth Clin Pharm. 1997;13:117–120.
2. Magder S, Lagonidis D. Effectiveness of albumin versus normal saline as a test of volume responsiveness in post-cardiac surgery patients. J Crit Care 1999;14:164-71.

# Ernest D, Belzberg AS, Dodek PM. Distribution of normal saline and 5% albumin infusions in cardiac surgical patients. Crit Care Med. 2001;29:2299-302.

# Zarro DL, Palanzo DA, Phillips TG. Albumin in the pump prime: its effect on postoperative weight gain. Perfusion. 2001;16:129-35.

1. Myers GJ, Legare JF, Sullivan JA, Leadon RB, Johnstone R, Swyer W, et al. Use of autologous blood as part of the perfusate for cardiopulmonary bypass: a priming technique. Perfusion. 2002;17:211-16.

# Arya VK, Nagdeve NG, Kumar A, Thingnam SK, Dhaliwal RS. Comparison of hemodynamic changes after acute normovolemic hemodilution using Ringer's lactate versus 5% albumin in patients on beta-blockers undergoing coronary artery bypass surgery. J Cardiothorac Vasc Anesth. 2006;20:812-8.

# Rex S, Scholz M, Weyland A, Busch T, Schorn B, Buhre W. Intra- and extravascular volume status in patients undergoing mitral valve replacement: crystalloid vs. colloid priming of cardiopulmonary bypass. Eur J Anaesthesiol 2006;23:1-9.

# Verheij J, van Lingen A, Beishuizen A, Christiaans HM, de Jong JR, Girbes AR, et al. Cardiac response is greater for colloid than saline fluid loading after cardiac or vascular surgery. Int Care Med. 2006; 32:1030-8.

1. Liou HL, Shih CC, Chao YF, Lin NT, Lai ST, Wang SH, et al. Inflammatory Response to Colloids Compared to Crystalloid Priming in Cardiac Surgery Patients with Cardiopulmonary Bypass. Chin J Physiol. 2012;55:210-18.
2. Skhirtladze K, Base EM, Lassnigg A, Kaider A, Linke S, Dworschak M, et al. Comparison of the effects of albumin 5%, hydroxyethyl starch 130/0.4 6%, and Ringer's lactate on blood loss and coagulation after cardiac surgery. Br J Anaesth. 2014;112:255-64.
