# Supplementary material for: Effect and safety of 4% albumin in the treatment of cardiac surgery patients: study protocol for the randomized, double-blind, clinical ALBICS (ALBumin In Cardiac Surgery) trial
Source: Trials. 2020 Feb 28;21:235. doi: 10.1186/s13063-020-4160-3 (PMC7048052; doi:10.1186/s13063-020-4160-3)
Supplement: Supplementary file 4 — Additional file 4. Ethics Committee approvals and Finnish Medical Agency (FIMEA) approvals. [file 13063_2020_4160_MOESM4_ESM.docx]

**Additional file 4: Ethical Committee approvals and Finnish Medical Agency (FIMEA) approvals**

| HOSPITAL DISTRICT OF HELSINKI AND UUSIMAA | Extract | 1 (1) |
| --- | --- | --- |
|  |  | Case number: HUS/2291/2016 |
|  |  | task: 13.02.01 |
| Ethics Committee, Department of Surgery | 06 Feb 2017 | Classified |

| Recipient | Eero Pesonen, PO Box 900, 00029 HUS |
| --- | --- |
| Trial title | Albumin in cardiac surgery - ALBICS |
| Reference | HUS/2291/2016 |
|  | Ethics Committee, Department of Surgery of the Hospital District of Helsinki and Uusimaa (HUS) reviewed your trial protocol in their meeting §286/14 Dec 2016 and requested some corrections to it. The Chair has been authorised by the Committee to approve the requested corrections and additional clarifications submitted to the Committee. |
|  | The corrections requested to be made to the protocol and the additional clarifications are specified in the documents submitted: |
|  | - E-mail, Eero Pesonen 03 Feb 2017 - Subject information leaflet, version 3, 30 Jan 2017 - Consent form, version 2, 30 Oct 2016 - Subject information leaflet and consent form in Swedish, version 3, 30 Jan 2017 - Trial protocol, version 3, 28 Nov 2016 - Description of the personal data file, version 3, 30 Jan 2017 - List of trial sites and investigators in Finland, version 3, 30 Jan 2017 - Statement regarding the quality of the trial facilities and the available equipment, version 3, 30 Jan 2017 - Statement regarding the eligibility of the person in charge of the trial, version 3, 30 Jan 2017 - Statement regarding trial fees and remuneration, version 3, 30 Jan 2017 - Insurance cover available for potential subjects in cases where patient insurance and pharmaceutical injuries insurance do not cover the trial, version 3, 30 Jan 2017 - Copy of opinion §286/14 Dec 2016 |
|  | The trial protocol and its appendices meet the requirements of Section 17, Subsection 3 of the Medical Research Act (488/1999). |
| Decision | The Chair decided to approve the requested corrections/additional clarifications and issue a favourable opinion on them on behalf of the Committee. |
|  | Corrections requested by the Committee; the opinion is free of charge (Ministry of Social Affairs and Health Decree 1417/2016, Section 1, Subsection 3). |
|  | In fidem, |
|  | Helsinki, 08 Feb 2017 |
|  | **[SIGNATURE]** |
|  | Leena-Maija Aaltonen |
|  | Chair |

| **Hospital District of Helsinki and Uusimaa** | **Opinion** | 1 (5) |
| --- | --- | --- |
| Ethics Committee, Department of Surgery | 14 Jun 2017 |  |

**Meeting information**

| **Time** | | | | | Wednesday 14 Jun 2017, at 01.04 - 03.41 pm | |
| --- | --- | --- | --- | --- | --- | --- |
| **Place** | | | | | Biomedicum 1, Conference Room 3, Floor P | |
| **Additional information** | | | | | Meeting of the Committee 7/2017 | |
| **Participants** | | | | | | |
| Present | | Leena-Maija Aaltonen, Chair, recused herself from §154  Leena Halme, Chair during §154  Eeva-Marja Sankila  Heikki Järvinen  Johan Marjamaa, left after §157  Janne Nikkinen  Jaana Vento  Gabriela Cedercreutz, Lay Member  Riitta Lehtonen, Deputy Lay Member | | | | |
| Other participants | | Minna Ruuska, Committee Secretary | | | | Secretary |
| Absent | | Micaela Hernberg  Kalevi Hietaniemi  Anna-Maria Koivusalo  Marjut Leidenius  Carita Sainio  Peter Raivio  Ulla Keränen  Lasse Svahnström | | | | |
| §169 | | Item No. HUS/2291/2016 | | | | |
|  | |  | | | | |
| **Trial title** | | | **Albumin in cardiac surgery - ALBICS; Protocol amendment** | | | |
| **Grounds for confidentiality** | | | Llt488/ §23; Matter relating to the Ethics Committee's opinion | | | |
| **Description** | | | A protocol amendment was submitted to the Committee for a review. The trial protocol was originally reviewed by the Ethics Committee on 26 Aug 2015, and a favourable opinion was issued on it.  This is a national, single-centre clinical drug trial comparing the use of albumin solution (Albuman, Sanquin, the Netherlands) and Ringer’s solution (Baxter) in the tubing of a cardiopulmonary bypass machine (i.e. priming) and as intra- and postoperative circulatory volume replacement solution.  The reported trial schedule is 1/2016 – 12/2019.  Version 4 of the protocol has now been submitted to the Committee for a review. | | | |
| **Delegation of review** | | | The National Committee on Medical Research Ethics (TUKIJA) has decided to delegate the review of the trial to the Ethics Committee of the Hospital District of Helsinki and Uusimaa (HUS) (delegation decision 09 Jun 2015, 104/06.00.00/2015, Ministry of Social Affairs and Health Decree on Clinical Drug Trials 841/2010, §2). | | | |
| **EudraCT number** | | | 2015-002556-27 | | | |
| **Person in charge of the trial** | | | | | | |
|  | | | Eero Pesonen, D.Med.Sc., Associate Professor, HUS | | | |
| **Investigators** | | | Eero Pesonen, D.Med.Sc., Associate Professor, Head of Department, HUS  Alexey Schramko, D.Med.Sc., HUS  Ville Pettilä, D.Med.Sc., Associate Professor, Chief Physician, HUS  Raili Suojaranta-Ylinen, D.Med.Sc., Associate Professor, Head of Department, HUS  Seppo Hiippala, D.Med.Sc., HUS  Erika Wilkman, D.Med.Sc., HUS  Kaapo Arvonen, Lic.Med., HUS  Ulla-Stina Salminen, D.Med.Sc., Associate Professor, Head of Department, HUS  Leo Ihlberg, D.Med.Sc., Associate Professor, HUS | | | |
| **Sponsor** | | | Helsinki University Hospital, Department of Anaesthesia, Intensive Care and Pain Medicine | | | |
| **Trial financier** | | | Helsinki University Hospital, Department of Anaesthesia, Intensive Care and Pain Medicine | | | |
| **Documents submitted** | | |  | | | |
|  | | | Cover letter, Eero Pesonen, 22 May 2017  Revisions to the protocol version 3 / 28 Nov 2016  Study Protocol version 4, 22 May 2017 | | | |
| **Decision proposal** | | | The Ethics Committee decides   1. on the matter in their meeting discussions; | | | |
|  | | | 1. to charge an opinion fee of €0 (Ministry of Social Affairs and Health Decree No. 1417/2016, §1 (3)). | | | |
| **Decision** | | | The Committee is of the opinion that the documents are in full compliance with the provisions of the Medical Research Act (488/99 and subsequent amendments) and the Medical Research Decree (986/99 and subsequent amendments), the data protection provisions, and the international obligations concerning medical research and the status of trial patients that medical research on human subjects must comply with.  The Ethics Committee decides   1. to issue a favourable opinion on the protocol amendment.   The Chair shall answer any questions concerning the factual content of the decision as necessary.   1. to charge the opinion fee as proposed.   The opinion issued by the Ethics Committee cannot be appealed. However, if the opinion of the Ethics Committee is unfavourable, the sponsor may bring the matter with similar contents before the Ethics Committee for reconsideration. At the request of the sponsor, the regional Ethics Committee must seek the opinion of the National Committee on Medical Research Ethics before issuing a second opinion on the matter (Medical Research Act 488/1999 and subsequent amendments).  A party liable for payment who considers that an error has occurred in determining the fee may claim rectification. Instructions for submitting a claim for rectification are appended. | | | |
| **Additional information** | | | Chair Leena-Maija Aaltonen, tel. +358 (0)50 427 1493  Committee Secretary Minna Ruuska, tel. +358 (0)50 427 9345  This extract from the uninspected minutes is hereby certified as genuine  In Helsinki, on 21 Jun 2017  **[SIGNATURE]**  Minna Ruuska  Secretary | | | |
| Sent for information | | | 21 Jun 2017 | | | |
| Annex | | | Instructions for submitting a claim for rectification | | | |
| Cc | | | Eero Pesonen, Peijas Hospital, PO Box 900, 00029 HUS | | | |

| HOSPITAL DISTRICT OF HELSINKI AND UUSIMAA | Opinion | 1 (1)  case number: HUS/2291/2016 |
| --- | --- | --- |
|  |  | task: 13.02.01 |
| Research Ethics Committees | 18 Jan 2019 | Classified |

Eero Pesonen

Albumin in cardiac surgery - ALBICS

| Reference | HUS/2291/2016  EudraCT number 2015-002556-27  Ethics Committee II of the Hospital District of Helsinki and Uusimaa (HUS) reviewed your trial protocol in their meeting §210 on 19 Dec 2018 and requested some corrections. The Chair has been authorised by the Committee to approve the requested corrections and additional clarifications submitted to the Committee.  The corrections and additional clarifications requested for the protocol are specified in the documents submitted: please see the annexes  The trial protocol and the appended documents meet the requirements of §17 (3) of the Medical Research Act (488/1999). |
| --- | --- |
| Decision | On behalf of the Committee, the Chair decided to approve the requested corrections/additional clarifications and issue a favourable opinion on them.  Corrections requested by the Committee, no opinion fee (§1 (3) of Decree 1287/2018 of the Ministry of Social Affairs and Health).  In fidem,  Helsinki  Lauri Tammilehto [SIGNATURE]  Chair |
| Annexes |  |
|  | HUS-2291-2016-17 Lausunto Eettinen toimikunta II 19.12.2018 §210 [Opinion of Ethics Committee II, 19 Dec 2018]  452940_313448_0.pdf  TVHn arvio muutosten eettisyydesta.pdf [Statement by the person in charge of the trial regarding the conformity of the amendments with research ethics] |

| 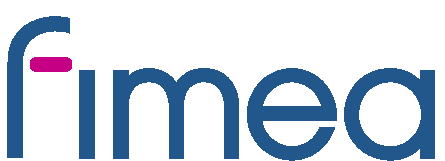 |  | **Notification of review** | | | 1(1) |
| --- | --- | --- | --- | --- | --- |
|  | | Helsinki, 25 Nov 2016 | KL No. | 136/ 2015 | |
|  |  |  | EudraCT | 2015-002556-27 | |

Hospital District of Helsinki and Uusimaa

Eero Pesonen

Peijas Hospital

PO Box 900

00029 HUS

Your clinical drug trial

**Comparison of the effect of 4% albumin and Ringers acetate in cardiopulmonary bypass (CPB) and intra- and postoperative volume replacement.**

**ALBICS**

The Finnish Medicines Agency Fimea (Senior Medical Officer Eeva Sofia Leinonen) reviewed your notification of the drug trial indicated in the subject line on 25 Nov 2016 and found no impediment to starting the trial. The trial may commence, provided that the Ethics Committee/TUKIJA has issued a favourable opinion on it.

The sponsor/investigator in charge of the trial must submit a report of the trial results to Fimea within a year of the end of the trial. The report should be submitted in the form of a summary, synopsis, offprint or another similar way. A comprehensive trial report containing the complete research results should be submitted to Fimea only upon request.

Finnish Medicines Agency Administrative Regulation No. 2/2012*, Clinical trials on medicinal products.*

Eija Mikkonen

Clinical Trials Coordinator

**Contact details:**

*Finnish Medicines Agency Fimea*

*Clinical Trials*

*PO Box 55*

*00034 FIMEA*

[*firstname.lastname@fimea.fi*](mailto:firstname.lastname@fimea.fi)

__________________________________________
_From: clinicaltrials@fimea.fi [clinicaltrials@fimea.fi]_
_Sent: 25 Jul 2017, 12.21 pm_
_To: Pesonen Eero_
_Subject: KL No. 136/2015 EudraCT 2015-002556-27_

_Notification of the review of a protocol amendment_
_Hospital District of Helsinki and Uusimaa_
_Eero Pesonen_

_Your clinical drug trial: ALBICS_
_Protocol amendment 22 May 2017_

_On 24 Jul 2017, the Finnish Medicines Agency (Fimea) reviewed the protocol amendment mentioned in the subject line._
_Fimea has no objection to the trial being continued in accordance with the amended protocol. The Medicines Act (395/87), §87 a._


_Suvi Mantere_
_Clinical Trials Coordinator_

_Contact details:_
_Finnish Medicines Agency Fimea_
_Clinical Trials_
_PO Box 55_
_00034 FIMEA_
_firstname.lastname@fimea.fi_
_###########################################_
_This message has been scanned by Trend Micro IMSS antivirus._

**From:** [clinicaltrials@fimea.fi](mailto:clinicaltrials@fimea.fi) <[clinicaltrials@fimea.fi](mailto:clinicaltrials@fimea.fi)>

**Sent:** Tuesday, 05 Mar 2019, 03.36 pm

**To:** Pesonen Eero

**Subject:** KL No. 136/2015 EudraCT 2015-002556-27

Notification of the review of a protocol amendment

Hospital District of Helsinki and Uusimaa

Eero Pesonen

Your clinical drug trial: ALBICS

Amendment 03 Sep 2018 version 5

On 04 Mar 2019, the Finnish Medicines Agency (Fimea) reviewed the protocol amendment mentioned in the subject line.

Fimea has no objection to the trial being continued in accordance with the amended protocol. The Medicines Act (395/87) §87 a.

Suvi Mantere

Clinical Trials Coordinator

Contact details:

Finnish Medicines Agency Fimea

Clinical Trials

PO Box 55

00034 FIMEA

[firstname.lastname@fimea.fi](mailto:firstname.lastname@fimea.fi)

###########################################

This message has been scanned by Trend Micro IMSS antivirus.

_Viestihistoria_
